# Multiple marginalized identities: Multilevel analysis of COVID-19 impacts on youth mental and behavioral health

**DOI:** 10.1016/j.pmip.2026.100178

**Published:** 2026-01-25

**Authors:** Jennifer D. Runkle, Kelsey Herbst, Sohpia C. Ryan, Martie P. Thompson, Margaret M. Sugg

**Affiliations:** a North Carolina Institute for Climate Studies, North Carolina State University, Asheville, NC, USA; b Department of Geography and Planning, Appalachian State University, Boone, NC, USA; c Department of Public Health, College of Health Sciences, Appalachian State University, Boone, NC, USA

**Keywords:** Youth mental health, Intersectionality, COVID-19 pandemic, Social determinants of health

## Abstract

**Objective:**

The COVID-19 pandemic exacerbated youth mental and behavioral health challenges in the United States, disproportionately impacting minoritized groups. Few studies examine how intersecting social determinants jointly shape these outcomes, particularly in underserved regions. This study applied an intersectional framework to assess pandemic-related changes and identify highest-risk strata.

**Methods:**

We applied a Multilevel Analysis of Individual Heterogeneity and Discriminatory Accuracy model to emergency department data (2010–2022) to estimate pre- and post-pandemic prevalence of depression, anxiety, self-harm, and attention-deficit/hyperactivity disorder among youth (ages 5–26) in western North Carolina (WNC) (n = 934,938) and statewide (n = 11,973,484). Intersectional strata were defined by COVID-19 period, sex, race/ethnicity, insurance type, and age. Predicted prevalence identified highest-risk strata for each outcome and region.

**Results:**

Lowest-risk strata were consistently pre-COVID, elementary-aged children with self-pay insurance, often male and from minoritized racial/ethnic groups. Highest-risk strata for depression and anxiety were post-COVID, young adult females with insurance across multiple racial/ethnic groups, with prevalence up to 11.9% (depression) and 12.9% (anxiety) in WNC. For self-harm, post-COVID adolescent females with insurance had the highest risk, with slightly higher prevalence in WNC (1.5%) than NC (1.4%). ADHD showed distinct patterns, with highest prevalence among male adolescents with Medicaid (7.1% in WNC, 11.4% statewide).

**Conclusions:**

Intersectional analysis revealed both shared and region-specific disparities. WNC showed higher top-risk prevalence for depression, anxiety, and self-harm, while statewide strata showed higher ADHD risk. Findings support regionally tailored prevention strategies, including rural workforce expansion, culturally responsive care, and standardized diagnostic practices.

## Introduction

The COVID-19 pandemic has exacerbated an unprecedented mental health crisis among U.S. youth, with emerging evidence pointing to wide disparities in psychological distress, suicidality, and behavioral health outcomes across sociodemographic groups [1–5]. The pandemic’s mental health impacts were not evenly distributed: adolescent girls experienced particularly sharp increases in depression and anxiety, while Black, Hispanic, and low-income youth faced disproportionate barriers to care and heightened psychological distress [4,6–9]. Other historical events, including the 1918–1919 influenza outbreak and the 2003 SARS epidemic, demonstrate that large-scale infectious disease events can exacerbate suicide risks and other mental health harms [10,11]. Yet the unparalleled scale, duration, and overlapping waves of disruption caused by the COVID-19 pandemic, including repeated surges, prolonged uncertainty, and coinciding social and economic crises, have amplified these risks. The convergence of school closures, economic uncertainty, grief, caregiver strain, and prolonged social isolation has disproportionately impacted youth from historically marginalized communities, where structural racism and longstanding inequalities in access to timely, culturally responsive care have exacerbated the psychological toll [12,13].

Racial and ethnic disparities in child and adolescent suicide risk have been widely documented [14–16]. Black, Hispanic, Asian American, and other youth of color face unique sociocultural risk factors, including experiences of discrimination, historical trauma, and racism-related stress, which are associated with heightened suicidality [16]. These gaps underscored the pre-pandemic trends showing an increase in suicide mortality among Black adolescents, from 2.55 per 100,000 in 2007 to 4.82 per 100,000 in 2017, with Black adolescents under age 13, particularly males, being twice as likely to die by suicide as White peers [17–21]. Despite this, access to culturally responsive mental health care remains limited [22], and the absence of a national mental health surveillance system hinders timely detection and prevention.

The COVID-19 pandemic has amplified these disparities [23]. The U. S. Centers for Disease Control and Prevention reported that emergency department visits for suspected suicide attempts among adolescents aged 12–17 years were 1.7 times higher in summer 2020 and 2.1 times higher in winter 2021 compared to corresponding periods in 2019 [24]. Our preliminary analyses, along with prior work using Crisis Text Line data, indicate elevated rates of suicidal ideation among children of essential workers, particularly youth with nonbinary gender identities, and a nearly threefold increase in suicide risk for Black children during the pandemic, underscoring the need to understand how intersecting social determinants shape youth mental health [25–27].

Most research on pandemic-related youth mental health outcomes have employed a single-axis analytic framework, which obscures the interactive effects of multiple social determinants. To address this limitation, the present study applies an intersectional multilevel analytic framework, Multilevel Analysis of Individual Heterogeneity and Discriminatory Accuracy (MAIHDA), to assess how intersecting social identities (e.g., race/ethnicity, sex, insurance payer, age group, and pre-/post-pandemic period) are associated with mental and behavioral health outcomes, including anxiety, depression, attention-deficit/hyperactivity disorder (ADHD), and self-harm. The literature is already overwhelmingly strong in documenting significant pre/post-pandemic increases in depression, anxiety, and self-harm among youth and young adults (e.g., (Racine et al., 2021; Madigan et al., 2023; Cheng et al., 2023; Hoyt et al., 2023). Our work builds on this foundation by taking a step further, using an intersectional modeling framework to uncover how these well-established mental-health impacts were distributed unequally across social identities.

The aim of this study is to identify intersectional strata of youth that exhibit disproportionately high or low predicted probabilities of mental and behavioral health outcomes before and after the COVID-19 pandemic. More specifically, we examined how risks for depression, anxiety, self-harm, and ADHD are distributed across intersecting dimensions of social identity and structural position. We also sought to describe how these intersectional risk patterns differ between Western North Carolina, a predominantly rural and medically underserved region, and the rest of the state. We hypothesize that risk will be unevenly distributed across intersectional strata, with elevated predicted probabilities for youth facing multiple structural disadvantages (e.g., rural residence, public insurance). MAIHDA extends conventional multilevel modeling by simultaneously estimating health risks across intersectional strata (e.g., White girls with public insurance during the post-pandemic period) and quantifying the variance attributable to these intersectional positions, thereby producing a more comprehensive understanding of population-level disparities [28,29]. This exploratory study applies an intersectional multilevel modeling framework to quantify differences in mental and behavioral health outcomes across combinations of social identities, rather than to establish causal relationships.

In our study region of western North Carolina (WNC), strong social cohesion and cultural resilience coexist with persistent behavioral health workforce shortages, limited specialty providers, and geographical barriers to care, particularly in more rural and mountainous communities. These structural constraints differ markedly from those in more urbanized and resource-rich areas of the state, making direct comparison between WNC and the state of North Carolina as a whole critical for identifying context-specific drivers of diverse risk and resilience. This comparative exploratory analysis sheds light on whether the intersectional social determinants influencing youth mental health operate similarly across settings or are amplified in rural, underserved geographies. This distinction is essential for informing state health policy, as resource allocation, workforce development, and service delivery models must be tailored to the unique needs of different regions. Findings will guide the design of targeted, equity-centered interventions, strengthen suicide prevention strategies for Black, Indigenous, and other youth of color, and support policy decisions aimed at mitigating both the immediate and long-term mental health consequences of COVID-19, thus enhancing preparedness for future public health crises.

## Methods

### Data

North Carolina emergency department (ED) admission data were obtained from the UNC Sheps Center for Health Services Research [30]. All diagnostic outcomes in this study were identified using International Classification of Diseases (ICD) codes documented at the time of the emergency department (ED) encounter. These codes are assigned by clinicians or trained medical coders based on clinical evaluations and presenting complaints, rather than through patient self-report or structured diagnostic interviews. Diagnostic codes from the International Classification of Diseases, Ninth and Tenth Revisions (ICD-9 and ICD-10, respectively) were used to identify four binary indicators of mental and behavioral health: (1) depression, (2) anxiety, (3), intentional self-harm, and (4) attention deficit hyperactivity disorder (ADHD) (Supplementary Table 1). We focused on these four diagnostic outcomes to capture the most prevalent and policy-relevant dimensions of youth mental and behavioral health during the COVID-19 pandemic.

These conditions have been consistently identified in global studies as those most affected by pandemic-related disruptions to daily life, schooling, and care access. A growing body of evidence shows increased prevalence of anxiety and depressive symptoms in youth worldwide [5], as well as sharp rises in pediatric cases of self-harm presenting to the emergency department [2,4]. Similarly, ADHD symptoms have been shown to worsen during the pandemic, likely reflecting changes in structure, stress, and learning or home environments [3]. Although ADHD is classified as a neurodevelopmental and externalizing disorder, it was included in our analysis given its high comorbidity with internalizing conditions and its sensitivity to psychological stressors. Together, these four outcomes provide a comprehensive view of the pandemic’s impact on youth emotional and behavioral dysregulation. We excluded substance misuse behaviors to maintain conceptual focus on internalizing and behavioral conditions that most directly reflect youth emotional distress. Substance use disorders differ etiologically and clinically from our list of primary outcomes, often co-occurring but arising from distinct pathways of risk, care-seeking, and diagnostic coding. Moreover, emerging evidence suggests that rates of youth substance use-related ED visits declined during the pandemic [31], further supporting our decision to focus on conditions that showed consistent global increases in prevalence.

ED admission records also included patient-level characteristics capturing patient admission date, gender, age, race/ethnicity, residential county, insurance payer, and county of admission. Emergency department visits, while imperfect, have been widely used as a proxy for population-level mental health burden and unmet need, particularly in youth [32,33]. For this analysis, we examined ED visits for patients aged 5 to 26 years occurring between July 16th, 2010, and September 30th, 2022, to capture over a decade of pre-pandemic trends and to provide sufficient follow-up through multiple waves of the COVID-19 pandemic. The study period ended on September 30, 2022, which represented the most recent fully validated data available, as each annual release includes records from the final quarter of the prior year through the first three quarters of the current year.

To examine regional disparities in mental and behavioral health, we compared patterns across the state of North Carolina and within a focused 18-county subregion in WNC. The WNC counties were defined according to the boundaries used by the WNC Health Network, which coordinates regional community health initiatives. This region is characterized as predominantly rural and medically underserved, with sociodemographic profiles and healthcare access patterns that differ from those in other parts of the state. The analytic sample includes 934,938 ED visits from WNC and 11,973,484 visits statewide.

### Social strata

Intersectional social strata were constructed using five individual-level characteristics recorded at the time of admission: sex (male, female), race/ethnicity (non-Hispanic [NH] White, non-Hispanic Black, Hispanic, non-Hispanic American Indian, non-Hispanic Asian/Native Hawaiian/Pacific Islander [Asian/NH/PI], and non-Hispanic Other), insurance payer type (self-pay, Medicaid, private, Other government/program-based), youth age group (children aged 5–11, adolescents aged 12–17, and young adults aged 18–26 years), and time period of their visit relative to the COVID-19 pandemic. Specifically, we included youth and young adults aged 5–26 years to align with the U.S. Department of Health and Human Services’ definition of “transitional-aged youth” (typically ages 16–26), a developmental group that experiences critical transitions in education, employment, and healthcare coverage [34]. Including this age range also reflects continuity in eligibility and insurance coverage patterns, as many individuals in this group remain on parental insurance programs through age 26 under the Affordable Care Act. Moreover, the inclusion of young adults captures a critical developmental window when mental and behavioral disorders often first emerge or intensify, thereby allowing us to assess early adult trajectories in the context of adolescent risk [35].

To account for changes in mental health trends associated with the pandemic, visits were classified as either pre-pandemic or during-/post-pandemic, with March 13, 2020, marking the U.S. national emergency declaration, used as the cut point [36]. ED records with missing information on key social strata predictors were excluded prior to formal analysis. This exclusion was expected, as social strata could only be constructed for observations with complete data on sex, race/ethnicity, insurance payer, age group, and admission date. The North Carolina dataset had 288 unique intersectional strata based on all possible combinations of these factors. None of the statewide strata contained fewer than 10 observations. When restricted to the 18-county region of WNC, 284 strata were initially identified. To ensure statistical reliability and robustness, strata with fewer than 10 observations were excluded from the WNC analysis (Supplementary Table 2), resulting in a final analytic sample of 261 strata. Details of strata size distributions and observation thresholds are provided in Table 2.

### Statistical analyses

This study employed an intersectional multilevel analysis of individual heterogeneity and discriminatory accuracy (MAIHDA) framework to examine disparities in mental and behavioral disorders among youth. Strata were defined by the intersection of sex, race/ethnicity, insurance type, age groupings, and COVID-19 time period [29]. Rather than evaluating these characteristics in isolation, the MAIHDA framework accounts for their joint influence, allowing for a more comprehensive assessment of how intersecting social identities shape the predicted risk of selected outcomes. Unlike standard multivariable regression, which models main effects and selected interaction terms, MAIHDA enables simultaneous estimation of all possible intersections among multiple social identities (e.g., race/ethnicity × sex × insurance × age × pandemic period) while preserving statistical power and avoiding model overparameterization. This approach quantifies both between-stratum variance (i.e., the extent to which differences in outcomes are explained by intersectional group membership) and within-stratum heterogeneity, thereby providing a measure of how much added explanatory value intersectional positions contribute beyond the additive effects of individual variables [28,29].

We used two-level generalized linear mixed models to examine our selected binary outcomes with individual ED visits nested within intersectional strata. Models specifying a binomial distribution with a logit link were estimated in R using the glmmTMB package. Each model included a random intercept for stratum to account for between-group variation tied to intersectional social characteristics. For the main analysis, only cases assigned to social strata were included, and a total of 21,979 cases could not be assigned to a stratum due to missing data. For each outcome, two models were fit. The baseline model (Model 2A) included the random intercept, acting as a baseline to assess how much of the variation was due to differences between strata. Model 2B added fixed effects for all five stratum-defining variables (sex, race/ethnicity, insurance type, age group, and COVID-19 period) as covariates; enabling assessment of the extent to which patient-level characteristics explained between-stratum variation. We assessed general intersectional effects using the Variance Partition Coefficient (VPC) to quantify the proportion of outcome variance attributable to differences across strata and the residual variance explained by additive effects, indicating the degree of group clustering and strata-level heterogeneity in outcomes; Proportional Change in Variance (PCV) to measure the reduction in between-stratum variance from Model 2A to Model 2B, showing how much of the stratified variability is explained by the inclusion of individual-level predictors; and the area under the curve (AUC), to evaluate each models’s discriminatory accuracy in distinguishing individuals with and without the outcome, where values closer to 1 indicate stronger predictive performance [37–39].

From the fixed-effects models, we derived adjusted odds ratios (aORs) and 95 % confidence intervals to quantify the average association between each social determinant of health and the likelihood of experiencing each outcome, controlling for all other variables. These aORs summarized additive effects across the full sample and are reported alongside variance metrics. To further examine disparities across intersectional strata, we generated predicted probabilities from Model 2B for each stratum, ranked them from highest to lowest risk, and identified the ten highest- and lowest-risk strata for each outcome. This ranking provides an interpretable insight into disparity patterns, highlighting which combinations of social positions were most strongly associated with mental and behavioral health-related ED visits. All analyses were conducted separately using R (version 4.3) [40] for the whole state of North Carolina and for an 18-county subregion in WNC. As a sensitivity analysis, we repeated all models using a shorter, more symmetrical analytic window, restricting data to two years before and approximately two years after the pandemic onset (March 13, 2018-September 30, 2022), to account for potential pre-pandemic secular trends. The extended baseline was retained as the main analysis because it provided greater statistical power, captured long-term pre-pandemic variability, and avoided bias introduced by incomplete post-pandemic data coverage.

This study was reviewed and approved by the Institutional Review Board at North Carolina State University (IRB#: 26747).

## Results

Descriptive analyses revealed notable geographic and demographic differences in the distribution of youth mental and behavioral health outcomes across North Carolina (Table 1). In western North Carolina (WNC), youth were disproportionately more likely to reside in rural areas (18.7 % in WNC vs. 4.1 % statewide), identify as non-Hispanic White (82.5 % vs. 46.7 %), and be insured through Medicaid (46.8 % vs. 43.4 %). Compared to the rest of the state, WNC had a slightly higher proportion of young adults aged 18–26 years (61.0 % vs. 59.7 %) and a lower proportion of children aged 5–11 (18.7 % vs. 20.7 %) presenting to the ED. Mental and behavioral health outcomes varied by region. The prevalence of anxiety and depression was higher among youth in WNC compared to North Carolina overall (anxiety: 5.7 % vs. 4.3 %; depression: 4.1 % vs. 3.2 %), while the prevalence of ADHD was slightly lower (2.2 % vs. 2.7 %). Rates of intentional self-harm were comparable across regions (0.4 %).

Table 3 provides a summary of fixed-effects logistic regression models for WNC and NC. In WNC, the post-COVID period was associated with significantly higher odds of depression (aOR: 1.42, 95 %CI: 1.38, 1.46), anxiety (aOR: 1.45, 95 %CI: 1.41, 1.48), self-harm (aOR: 1.27, 95 %CI: 1.17, 1.38), and ADHD (aOR: 1.20, 95 %CI: 1.15, 1.24) diagnosis. Females had a higher odds of depression and anxiety, but lower odds of ADHD compared to males. Compared to non-Hispanic White youth, non-Hispanic Black, Hispanic, and American Indian youth generally had lower odds of depression and anxiety. However, American Indian youth exhibited elevated odds for self-harm (aOR:1.26, 95 %CI: 1.15, 1.37) and Asian youth showed a higher odds of depression (aOR:1.25, 95 %CI: 1.08, 1.46). Adolescents (12–17 years) and young adults (18–26 years) had markedly higher odds of depression, anxiety, and self-harm compared to children, with adolescents exhibiting the highest odds for self-harm (aOR: 18.96, 95 %CI: 14.80, 24.28). For the state of NC more broadly, similar patterns were observed with a few notable exceptions. The post-COVID period was associated with slightly higher odds across all outcomes compared to the pre-COVID period. Non-Hispanic Black, Hispanic, and American Indian youth generally had lower odds of all four conditions compared to non-Hispanic White youth, with one exception Non-Hispanic Asian youth had higher odds of self-harm (aOR: 1.16, 95 %CI: 1.06, 1.28).

Intersectional risk strata for WNC and NC are detailed in Table 4. Supplementary figures S3 and S4 display plotted model-derived predicted proportions for unique strata combinations for WNC and the full state of North Carolina, respectively.

### Depression

For both WNC and NC, the intersectional strata at the lowest predicted risk of depression were pre-COVID, male, elementary-aged (5–11 years) children across racial and ethnic groups with self-pay insurance, with prevalence estimates less than 0.4 %. The highest risk strata in both regions were post-COVID, female, young adults (18–26 years), with predicted prevalence ranging from 10.5 % to 11.9 % in WNC and 10.0 % to 11.4 % in NC.

### Anxiety

Again, we note that in WNC and NC, the strata with the lowest predicted anxiety prevalence were pre-COVID, male, age 5–11 years across multiple racial and ethnic groups with self-pay insurance, with prevalence estimates between 0.4 % and 0.6 %. The highest-risk strata in WNC were post-COVID, female, young adults across NH Asian/NH/PI and “Other” racial/ethnic groups with Medicaid, private, or “other” insurance coverage, showing predicted prevalence between 11.5 % and 12.9 %. In NC, the highest-risk strata were also post-COVID, female, young adults with “Other” or private insurance, across White, Asian/NH/PI, Hispanic and Black groups, with prevalence ranging from 10.4 % to 11.6 %.

### Self-harm

The lowest predicted prevalence of self-harm was observed in pre-COVID, children, with self-pay insurance for both males and females identifying as Non-Hispanic Black and Hispanic in WNC and predominantly males identifying as Non-Hispanic Black and American Indian in NC, with the predicted prevalence estimates of 0.0 % across all five lowest strata. The highest-risk strata in WNC were post-COVID, adolescent females with Medicaid, private, or “Other” insurance coverage, across non-Hispanic White, Non-Hispanic Asian/NH/PI, and American Indian groups. Predicted prevalence ranged from 1.2 % to 1.5 %. In NC, the highest-risk strata were similarly concentrated in post-COVID, adolescent females with Medicaid, private, or “Other” insurance, across non-Hispanic White and Non-Hispanic Asian/NH/PI adolescents, with a predicted prevalence between 1.3 % and 1.4 %.

### ADHD

The strata with the lowest predicted ADHD prevalence were predominantly female young adults (18–26 years) or children with self-pay insurance, across American Indian and Hispanic youth in WNC and across Non-Hispanic Asian/NH/PI, Hispanic, and American Indian youth in NC, with prevalence estimates between 0.1 % and 0.3 %. The highest-risk strata in WNC were male adolescents with Medicaid, across non-Hispanic White, Black, and “other” racial/ethnic groups, with predicted prevalence ranging from 5.6 % to 7.1 %. The highest prevalence was among non-Hispanic White adolescent males with Medicaid (7.0 %), followed by non-Hispanic Black males with Medicaid post-COVID (6.8 %). For NC, the highest-risk strata were also male adolescents, but with a broader spread of insurance types (e.g., Medicaid, private, and “Other”), and higher predicted prevalence ranging from 10.2 and 11.4 %. The highest prevalence was among non-Hispanic White adolescent males with Medicaid pre-COVID (11.4 %), followed by the same group post-COVID (11.1 %).

### Sensitivity analysis

Although we could not include a full two years of post-pandemic data due to the timing of data availability, results from the truncated, more symmetrical period were highly consistent with those from the primary model using the longer baseline period (Supplementary Tables 5-7).

## Discussion

Our study employed an analytic intersectional approach to examine the prevalence in anxiety, depression, self-harm, and ADHD for young people before and after the COVID-19 pandemic. Results revealed that the lowest-predicted prevalence for all outcomes was consistently observed among pre-COVID, elementary-aged (5–11 years) children with self-pay insurance, most often male and from minoritized groups. The highest-risk strata for depression and anxiety were consistently post-COVID, young adult females with non-traditional (self-pay) and traditional insurance coverage (“Other”, Medicaid, Private), spanning multiple racial and ethnic groups. However, predicted prevalence for these top-risk strata were often higher in WNC than in NC, which may reflect the influence of rurality, behavioral health workforce shortages, and insurance instability that could compound vulnerability in this largely rural region.

For self-harm, both WNC and NC showed the greatest risks among post-COVID adolescent females with non-self-pay insurance, but WNC exhibited a higher prevalence and was more likely to include American Indian youth, reflecting the local population composition and potentially unique service access challenges. We observed a different pattern for ADHD, with the highest prevalence in male adolescents with Medicaid, particularly non-Hispanic White and Black youth. Results revealed that state-wide rates in top-risk strata exceeded those in WNC, suggesting that urban and suburban areas of NC may have greater diagnostic capacity or different thresholds for ADHD diagnosis. Findings further demonstrate that while high- and low-risk intersectional profiles are broadly consistent across the state, regional variation in the magnitude and composition of these risks likely reflects differences in healthcare infrastructure and workforce, access, insurance coverage patterns, and population demographics.

Our results also revealed pronounced sex-based differences in predicted prevalence across health outcomes following the onset of COVID-19. After the pandemic, females, particularly young adults for depression and anxiety and adolescents for self-harm, consistently appeared in the highest-risk strata, whereas males were predominantly featured in the top-risk strata for ADHD, especially adolescent males with Medicaid. These findings align with existing literature documenting higher rates of internalizing disorders, such as depression and anxiety, among adolescent and young adult females. This gendered pattern is perceived to be driven by biological factors like hormonal fluctuations during puberty and gendered psychosocial stressors, including increased exposure to care-giving burdens, social isolation, and appearance-related pressures exacerbated by the pandemic [41,42]. Conversely, higher ADHD prevalence among males is consistent with well-established patterns of overrepresentation of males in ADHD diagnosis, potentially reflecting neurodevelopmental differences and gender differences in symptom recognition, with hyperactive-impulsive presentations rather than trouble paying attention more likely to be detected in male children [43]. This intersectional analysis extends prior work by demonstrating that these sex-based differences persist and, in some cases, intensify when examined alongside other social determinants such as insurance type, race/ethnicity, and age, highlighting the importance of this approach to understanding pandemic-related shifts in youth mental and behavioral health.

Our results show that pandemic-related shifts in youth mental and behavioral health were concentrated within specific intersections of age, sex, race/ethnicity, and insurance type. MAIHDA is emerging as a quantitative method that improves upon standard approaches by revealing patterns among multiple vulnerable social identities that would otherwise remain masked. A growing body of research demonstrates the importance of an intersectional approach in the context of COVID-19, revealing that youth characterized by multiple marginalized identies experienced the largest increase in depression, anxiety, and suicide-related diagnoses, particularly Hispanic, Asian, and Black females [23,44]. National and international studies further show that intersecting dimensions of race, gender, geography, and sexuality compounded vulnerability to pandemic-related stress, with rural women of color and sexual minority youth facing disproportionate psychological distress. [40,45,46]. International intersectional-based research similarly points to disproportionate pandemic-era burdens among sexual minority women and young adults, and documents how risk profiles shift across the life course and social positions [47–49]. Taken together, this evidence demonstrates how intersectional methods are critical for identifying which combinations of social positions place youth at the greatest risk and for understanding how distinct patterns of risk evolve following large-scale crises.

The COVID-19 pandemic should be understood not only as a public health emergency but as a profound socio-economic and political disruption that will likely have enduring consequences for this generation of young people. Its disruption to education, family stability, and access to care has produced acute shocks to youth mental and behavioral health, and may be shaping cohort effects that could reverberate across the life course [5,50]. For young people, particularly those navigating intersecting vulnerabilities of race, class, geography, and gender, the pandemic appears to have left an imprint that could entrench disparities well into adulthood [51,52]. COVID-19 demonstrated that mental health trajectories are inseparable from broader social and political structures [53], and that resilience likely depends on equitable investment in youth during times of crisis [54,55].

Findings on high-risk intersectional strata can be used to inform prevention and tailored screening or treatment strategies. The elevated prevalence of depression and anxiety in young adult females with unstable or self-pay insurance coverage college-based stepped-care and proactive screening models that expand access and continuity of care for financially insecure students [56]. Our results identifying a higher predicted prevalence of self-harm in American Indian adolescent females in WNC provide an opportunity to guide culturally grounded, community-based behavioral health programs co-developed with tribal partners. Similar programs, including the White Mountain Apache community-based suicide surveillance and brief intervention system and the Family Spirit home-visiting program, have reduced self-injury and improved behavioral health in the context of tribal partnerships and multi-tiered individual, family, and community-level interventions [57,58]. At the statewide level, concentration of ADHD risk among Medicaid-insured adolescent males points to opportunities for evidence-based behavioral management and integrated pediatric care models consistent with the American Academy of Pediatrics guideline, including care coordination and parent-training within primary care [59]. More broadly, an intersectionality-informed approach provides a policy framework to target resources toward strata facing compounded disadvantage, aligning service design with structural realities rather than single-axis risks [60]..

### Strengths

One key strength of this study is the use of the MAIHDA, which extends traditional multilevel models to quantify both between- and within-stratum variation to allow for a robust examination of how intersecting social identities jointly shape health outcomes [28,29]. This approach avoids the statistical inefficiencies and interpretative limitations typically encountered when using multiple interaction terms in standard regression models [61], enabling simultaneous estimation of all possible identity combinations while retaining statistical power. The comparison of predicted prevalence across intersectional strata facilitates nuanced identification of low- and high-risk groups [39]. Additionally, comparing results between WNC and the state of NC enhances external validity for regional policy planning and allows assessment of how structural determinants operate across geographical contexts and regions [62].

#### Limitations

Despite these strengths, MAIHDA models are largely descriptive and explanatory in nature, but do not inherently infer a causal relationship, as observed associations cannot establish temporal ordering or rule out unmeasured confounding [29]. Use of administrative health data may also introduce ascertainment bias, as some individuals with limited access to healthcare due to socioeconomic factors, insurance coverage, or geographical location may be underrepresented [63]. Another limitation includes our ED data are encounter-based and do not include longitudinal patient identifiers; whereby multiple visits from the same individual cannot be linked over time. ED data reflect patterns of healthcare use, access, and coding practices rather than true disorder incidence and our findings should be interpreted as indicators of mental and behavioral health service use rather than underlying prevalence or first onset. Lastly, our analysis assumes that the chosen social identity dimensions sufficiently capture the relevant axes of structural barriers; not including factors like disability status, sexual orientation, or immigration status may inadvertently underestimate the complexity of intersectional identities [60,61].

#### Summary

Our findings demonstrate the need for regionally tailored mental and behavioral health strategies. In WNC, higher prevalence of depression, anxiety, and self-harm among high-risk strata suggests the importance of expanding the rural behavioral health workforce, improving insurance stability, and increasing access to culturally responsive services for diverse groups, including American Indian youth. Conversely, higher ADHD prevalence in statewide top-risk strata points to the need for standardized screening and diagnostic practices across regions to ensure optimal identification and treatment. Targeted resource allocation that reflects these geographic and population-specific patterns will be critical to reducing disparities and strengthening the state’s youth mental health response.

## Supplementary Material

suppl material

## Figures and Tables

**Table 1:** Descriptive statistics of individual observations in the state of North Carolina (N = 11,973,484; intersectional strata = 288) and the subregion of Western North Carolina (N = 934,938; intersectional strata = 261).

	North Carolinan (%)	Western North Carolinan (%)

Age		
Children (5–11)	2,472,771 (20.7)	175,048 (18.7)
Adolescents (12–17)	2,347,447 (19.6)	189,408 (20.3)
Young Adults (18–26)	7,153,266 (59.7)	570,482 (61.0)
Sex		
Male	5,039,798 (42.1)	402,542 (43.1)
Female	6,933,686 (57.9)	532,396 (56.9)
Race/Ethnicity		
Non-Hispanic White	5,596,896 (46.7)	771,736 (82.5)
Non-Hispanic Black or African American	4,597,407 (38.4)	76,365 (8.2)
Hispanic	1,089,860 (9.1)	50,260 (5.4)
Non-Hispanic American Indian	183,898 (1.5)	8,590 (0.9)
Non-Hispanic Asian/Native Hawaiian/Pacific Islander	74,909 (0.6)	3,638 (0.4)
Non-Hispanic Other	430,514 (3.6)	24,349 (2.6)
Insurance Payer		
Self-Pay	2,908,676 (24.3)	213,279 (22.8)
Medicaid	5,201,078 (43.4)	437,628 (46.8)
Private Insurer	3,175,868 (26.5)	243,658 (26.1)
Other	687,862 (5.7)	40,373 (4.3)
COVID-19 Period		
Pre-COVID	10,306,546 (86.1)	807,856 (86.4)
Post-COVID	1,666,938 (13.9)	127,082 (13.6)
Urbanity		
Urban	9,623,338 (80.4)	505,234 (54.0)
Suburban	1,861,095 (15.5)	254,825 (27.3)
Rural	489,051 (4.1)	174,879 (18.7)
Mental & Behavioral Health Outcomes		
Depression	385,646 (3.2)	38,209 (4.1)
Anxiety	516,573 (4.3)	53,695 (5.7)
Intentional Self-Harm	48,564 (0.4)	4,012 (0.4)
ADHD*	327,862 (2.7)	20,807 (2.2)

* ADHD = Attention-deficit/hyperactivity disorder.

**Table 2:** Intersectional strata counts and proportions meeting observation thresholds across North Carolina and western North Carolina subregion.

Observation Per Stratum	Western North Carolina N (%)	North Carolina N (%)

100 or more	180 (69.0 %)	277 (96.2 %)
50 or more	212 (81.2 %)	286 (99.3 %)
30 or more	231 (88.5 %)	288 (100.0 %)
20 or more	247 (94.6 %)	288 (100.0 %)
10 or more	261 (100.0 %)	288 (100.0 %)

**Table 3:** Adjusted odds ratios and summary statistics for youth mental and behavioral health outcomes by intersectional predictors in North Carolina and western North Carolina subregion.

	WNC				NC			
	aOR^a^ (95 % CI)^b^				aOR (95 % CI)			
	Depression	Anxiety	Self-Harm	ADHD	Depression	Anxiety	Self-Harm	ADHD

*COVID-19*								
Pre-COVID (Ref)	–	–	–	–	–	–	–	–
Post-COVID	1.42 (1.38, 1.46)	1.45 (1.41, 1.48)	1.27 (1.17, 1.38)	1.20 (1.15, 1.24)	1.42 (1.41, 1.43)	1.56 (1.55, 1.57)	1.04 (1.02, 1.07)	1.35 (1.33, 1.36)
*Sex*								
Male (Ref)	–	–	–	–	–	–	–	–
Female	1.30 (1.27, 1.33)	1.22 (1.20, 1.24)	1.00 (0.94, 1.07)	0.43 (0.42, 0.45)	1.40 (1.39, 1.41)	1.32 (1.32, 1.33)	1.14 (1.12, 1.16)	0.44 (0.44, 0.46)
*Race/Ethnicity*								
NH White (Ref)	–	–	–	–	–	–	–	–
NH Black	0.76 (0.73, 0.80)	0.72 (0.69, 0.74)	0.79 (0.74, 085)	0.93 (0.88, 0.98)	0.47 (0.46, 0.47)	0.43 (0.42, 0.43)	0.45 (0.44, 0.46)	0.56 (0.55, 0.56)
Hispanic	068 (0.65, 0.72)	0.67 (0.64, 0.71)	0.70 (0.65, 0.77)	0.36 (0.33, 0.39)	0.57 (0.56, 0.58)	0.53 (0.53, 0.54)	0.66 (0.63, 0.68)	0.28 (0.28, 0.29)
NH American Indian	0.66(0.58, 0.75)	0.61 (0.55, 0.69)	1.26 (1.15, 1.37)	025 (019, 0.32)	0.42 (0.40, 0.43)	0.44 (0.43, 0.45)	0.54 (0.50, 0.59)	0.35 (0.33, 0.36)
NH Asian/NH/PI	1.25 (1.08, 1.46)	1.04 (0.91, 1.20)	1.18 (0.93, 1.49)	0.57 (0.44, 0.76)	0.81 (0.78, 0.85)	0.64 (0.61, 066)	1.16 (1.06, 1.28)	0.26 (0.24, 0.28)
Other	0.77 (0.71, 0.83)	0.61 (0.57, 0.68)	1.05 (0.95, 1.16)	0.75 (0.68, 0.83)	0.72 (0.71, 0.73)	0.64 (0.63, 0.65)	0.95 (0.91, 0.99)	0.67 (0.65, 0.68)
*Insurance Payer*								
Self-Pay	0.70 (0.68, 0.72)	0.75 (0.74, 0.77)	0.79 (0.72, 0.87)	0.61 (0.58, 0.64)	0.64 (0.63, 0.65)	068 (067, 0.68)	0.74 (0.72, 076)	0.54 (0.53, 0.55)
Medicaid	1.09 (1.06, 1.12)	1.10 (1.08, 1.13)	0.95 (0.88, 1.02)	1.72 (1.66, 1.78)	0.97 (0.96, 0.98)	0.96 (0.95, 0.97)	0.86 (0.84, 0.88)	1.58 (1.57, 1.59)
Private Insurer (Ref)	–	–	–	–	–	–	–	–
Other	1.12 (1.06, 1.17)	1.08 (1.03, 1.12)	1.09 (0.94, 1.27)	1.47 (1.37, 1.58)	1.10 (1.09, 1.12)	0.96 (0.95, 0.98)	1.15 (1.11, 1.19)	1.12 (1.10, 1.14)
*Age Group*								
Children 5–11 (Ref)	–	–	–	–	–	–	–	–
Adolescents (12–17)	11.25 (10.43, 12.12)	4.37 (4.16, 4.59)	18.96 (14.80, 24.28)	1.29 (1.24, 1.34)	12.09 (11.83, 12.35)	4.76 (4.69, 4.83)	16.73 (15.79, 17.72)	1.52 (1.51, 1.53)
Young Adults (18–26)	12.98 (12.06, 13.98)	7.37 (7.04, 7.72)	10.88 (8.58, 13.81)	0.66 (0.63, 0.68)	10.48 (10.26, 10.70)	6.26 (6.18, 6.35)	7.62 (7.19, 8.07)	0.56 (0.56, 0.57)
Variance Partition Coefficient	13.0 %	3.8 %	3.8 %	3.5 %	15.05	7.8 %	4.9 %	2.7 %
Proportional Change in Variance	61.1 %	84.9 %	88.4 %	84.8 %	62.8 %	72.0 %	88.6 %	89.3 %
Area Under Receiver Operating Characteristic Curve	0.67	0.66	0.68	0.70	0.71	0.70	0.73	0.74

a Adjusted odds ratios (aOR) from multivariable logistic regression models (Model 2B). Each estimate is adjusted for all other predictors in the model (COVID-19 period, sex, race/ethnicity, insurance type, and age group). Reference groups indicated in the table.

b Confidence interval.

**Table 4:** Predicted prevalence of youth mental and behavioral health outcomes: five highest- and lowest-ranked strata in North Carolina and western North Carolina.^a^ Strata were defined by COVID-19 period, sex, race/ethnicity, insurance type, and age group. The five lowest- and five highest-ranked strata for each outcome were identified based on predicted prevalence, separately for North Carolina and western North Carolina.

Rank	COVID-19 Perio	Sex	Race/Ethnicity	Insurance Payer	Age Group	n	Predicted Prevalence (%)

**WNC**^a^ **– Depression**							
*5 Lowest* 1	Pre	Male	NH American Indian	Self-Pay	Children (5–11)	40	0.3
2	Pre	Male	NH Other	Self-Pay	Children (5–11)	158	0.3
3	Pre	Male	NH Asian/NH/PI	Self-Pay	Children (5–11)	21	0.4
4	Pre	Male	Hispanic	Self-Pay	Children (5–11)	488	0.4
5	Pre	Male	NH American Indian	Medicaid	Children (5–11)	902	0.4
*5 Highest* 257	Post	Female	NH Other	Other	Young Adult (18–26)	21	10.5
258	Post	Female	NH Asian/NH/PI	Other	Young Adult (18–26)	19	10.9
259	Post	Female	Hispanic	Other	Young Adult (18–26)	213	11.1
260	Post	Female	NH Black	Other	Young Adult (18–26)	214	11.8
261	Post	Female	NH White	Other	Young Adult (18–26)	2042	11.9
**NC – Depression**							
*5 Lowest* 1	Pre	Male	NH American Indian	Self-Pay	Children (5–11)	1091	0.2
2	Pre	Male	NH Other	Self-Pay	Children (5–11)	3944	0.3
3	Pre	Male	NH Asian/NH/PI	Self-Pay	Children (5–11)	545	0.3
4	Pre	Male	Hispanic	Self-Pay	Children (5–11)	13,360	0.3
5	Pre	Male	NH Black	Self-Pay	Children (5–11)	25,819	0.3
*5 Highest* 284	Post	Female	NH Other	Other	Young Adult (18–26)	1423	10.0
285	Post	Female	NH Asian/NH/PI	Other	Young Adult (18–26)	431	10.4
286	Post	Female	Hispanic	Other	Young Adult (18–26)	5860	11.0
287	Post	Female	NH Black	Other	Young Adult (18–26)	18,129	11.2
288	Post	Female	NH White	Other	Young Adult (18–26)	22,170	11.4
**WNC – Anxiety**							
*5 Lowest* 1	Pre	Male	NH Other	Self-Pay	Children (5–11)	158	0.4
2	Pre	Male	NH American Indian	Self-Pay	Children (5–11)	40	0.4
3	Pre	Male	Hispanic	Self-Pay	Children (5–11)	488	0.4
4	Pre	Male	NH Black	Self-Pay	Children (5–11)	280	0.5
5	Pre	Female	NH Other	Self-Pay	Children (5–11)	148	0.5
*5 Highest* 257	Post	Female	NH Asian/NH/PI	Other	Young Adult (18–26)	19	11.5
258	Post	Female	NH Other	Medicaid	Young Adult (18–26)	14,586	12.4
259	Post	Female	NH Asian/NH/PI	Medicaid	Young Adult (18–26)	68	12.8
260	Post	Female	NH Other	Private	Young Adult (18–26)	14,817	12.9
261	Post	Female	NH Asian/NH/PI	Private	Young Adult (18–26)	122	12.9
**NC – Anxiety**							
*5 Lowest* 1	Pre	Male	NH American Indian	Self-Pay	Children (5–11)	1091	0.5
2	Pre	Male	NH Other	Self-Pay	Children (5–11)	3944	0.5
3	Pre	Male	NH Asian/NH/PI	Self-Pay	Children (5–11)	545	0.5
4	Pre	Male	NH American Indian	Medicaid	Children (5–11)	12,983	0.6
5	Pre	Male	Hispanic	Self-Pay	Children (5–11)	13,360	0.6
*5 Highest* 284	Post	Female	NH White	Private	Young Adult (18–26)	134,706	10.4
285	Post	Female	NH Asian/NH/PI	Other	Young Adult (18–26)	431	10.5
286	Post	Female	Hispanic	Other	Young Adult (18–26)	5860	11.1
287	Post	Female	NH Black	Other	Young Adult (18–26)	18,129	11.4
288	Post	Female	NH White	Other	Young Adult (18–26)	22,170	11.6
**WNC – Self-Harm**							
*5 Lowest* 1	Pre	Male	NH Black	Self-Pay	Children (5–11)	280	0.0
2	Pre	Male	Hispanic	Self-Pay	Children (5–11)	488	0.0
3	Pre	Female	NH Black	Self-Pay	Children (5–11)	258	0.0
4	Pre	Female	Hispanic	Self-Pay	Children (5–11)	408	0.0
5	Post	Male	NH Black	Self-Pay	Children (5–11)	33	0.0
*5 Highest* 257	Post	Female	NH White	Other	Adolescent (12–17)	268	1.2
258	Post	Female	NH Asian/NH/PI	Private	Adolescent (12–17)	37	1.2
259	Post	Male	NH American Indian	Private	Adolescent (12–17)	10	1.3
260	Post	Female	NH American Indian	Medicaid	Adolescent (12–17)	144	1.4
261	Post	Female	NH American Indian	Private	Adolescent (12–17)	14	1.5
**NC – Self-Harm**							
*5 Lowest* 1	Pre	Male	NH Black	Self-Pay	Children (5–11)	25,819	0.0
2	Pre	Male	NH American Indian	Self-Pay	Children (5–11)	1091	0.0
3	Post	Male	NH Black	Self-Pay	Children (5–11)	5181	0.0
4	Post	Male	NH American Indian	Self-Pay	Children (5–11)	115	0.0
5	Pre	Female	NH Black	Self-Pay	Children (5–11)	24,325	0.0
*5 Highest* 284	Post	Female	NH Asian/NH/PI	Medicaid	Adolescent (12–17)	471	1.3
285	Post	Female	NH White	Medicaid	Adolescent (12–17)	35,281	1.4
286	Post	Female	NH White	Other	Adolescent (12–17)	5073	1.4
287	Post	Female	NH Asian/NH/PI	Private	Adolescent (12–17)	833	1.4
288	Post	Female	NH White	Private	Adolescent (12–17)	40,100	1.4
**WNC – ADHD**							
*5 Lowest* 1	Pre	Female	NH American Indian	Self-Pay	Young Adult (18–26)	921	0.1
2	Pre	Female	NH American Indian	Self-Pay	Children (5–11)	31	0.1
3	Post	Female	NH American Indian	Self-Pay	Young Adult (18–26)	82	0.2
4	Pre	Female	Hispanic	Self-Pay	Young Adult (18–26)	3603	0.2
5	Pre	Female	NH American Indian	Self-Pay	Adolescent (12–17)	53	0.2
*5 Highest* 257	Post	Male	NH Other	Medicaid	Adolescent (12–17)	62	5.6
258	Pre	Male	NH Black	Medicaid	Adolescent (12–17)	3972	5.7
259	Pre	Male	NH White	Medicaid	Adolescent (12–17)	38,180	6.0
260	Post	Male	NH Black	Medicaid	Adolescent (12–17)	561	6.8
261	Post	Male	NH White	Medicaid	Adolescent (12–17)	4819	7.1
**NC – ADHD**							
*5 Lowest* 1	Pre	Female	NH Asian/NH/PI	Self-Pay	Young Adult (18–26)	4310	0.2
2	Pre	Female	Hispanic	Self-Pay	Young Adult (18–26)	98,133	0.2
3	Pre	Female	NH American Indian	Self-Pay	Young Adult (18–26)	17,924	0.2
4	Post	Female	NH Asian/NH/PI	Self-Pay	Young Adult (18–26)	805	0.2
5	Pre	Female	NH Asian/NH/PI	Self-Pay	Children (5–11)	440	0.3
*5 Highest* 284	Post	Male	NH White	Private	Adolescent (12–17)	33,698	10.2
285	Post	Male	NH Other	Medicaid	Adolescent (12–17)	1827	10.4
286	Post	Male	NH White	Other	Adolescent (12–17)	3925	10.5
287	Pre	Male	NH White	Medicaid	Adolescent (12–17)	224,740	11.4
288	Post	Male	NH White	Medicaid	Adolescent (12–17)	26,370	11.1

a WNC = western North Carolina.

## Appendix A. Supplementary data

Supplementary data to this article can be found online at https://doi.org/10.1016/j.pmip.2026.100178.
