# Supplementary material for: Multiple marginalized identities: Multilevel analysis of COVID-19 impacts on youth mental and behavioral health
Source: Pers Med Psychiatry. Author manuscript; Available in PMC 2026 May 7. (PMC13148411; doi:10.1016/j.pmip.2026.100178)
Supplement: suppl material [file NIHMS2164687-supplement-suppl_material.pdf]

S1 Table. International Classifications of Diseases, Ninth and Tenth Revision, Clinical Modification (ICD-9-CM) and ICD-10-CM) codes used to define youth mental and behavioral disorders.

**Mental and Behavioral Disorders**	**ICD-9 Codes**	**ICD-10 Codes**
Depression	296.2, 296.3, 300.4, 311, 296.82	F32, F33, F34.1, F38.1
Anxiety	293.84, 300.0, 300.2, 300.3, 301, 308, 309.8	F06.4, F40, F41, F42, F43.0, F43.1, F43.22, F43.8, F48.8, R45.7
Intentional Self-Harm	E95	X6, X7, X8, T14.91
ADHD*	314	F90.X, R41.840

*ADHD=Attention-deficit/hyperactivity disorder

S2 Table. Characteristics of youth excluded from the analytic sample for the subregion of western North Carolina due to inclusion in strata with fewer than 10 observations (n=95).

	**Non-Analyzed Sample**<br>**n/N (%)**
Dimensions of Social Position	
Age	
Children (5-11)	56/95 (58.9)
Adolescents (12-17)	39/95 (41.1)
Young Adults (18-26)	0/95 (0.0)
Sex	
Male	43/95 (45.3)
Female	52/95 (54.7)
Race/Ethnicity	
NH White	0/95 (0.0)
NH Black or African American	0/95 (0.0)
Hispanic	0/95 (0.0)
NH American Indian	43/95 (45.3)
NH Asian/Native Hawaiian/Pacific Islander	32/95 (33.7)
NH Other	20/95 (21.1)
Insurance Payer	
Self-Pay	42/95 (44.2)
Medicaid	0/95 (0.0)
Private Insurer	12/95 (12.6)
Other	41/95 (43.2)
COVID-19 Period	
Pre-COVID	16/95 (16.8)
Post-COVID	79/95 (83.2)
**Outcomes**	
Depression	3/95 (3.2)
Anxiety	10/95 (10.5)
Intentional Self-Harm	0/95 (0.0)
*ADHD**	0/95 (0.0)

*ADHD=Attention-deficit/hyperactivity disorder

S3 Figure. Predicted proportions of youth mental and behavioral health outcomes across intersectional social strata (sex, race/ethnicity, insurance type, age group, and COVID-19 period) within the western North Carolina (WNC) subregion. Every 10th ranked stratum combination is displayed for interpretability.

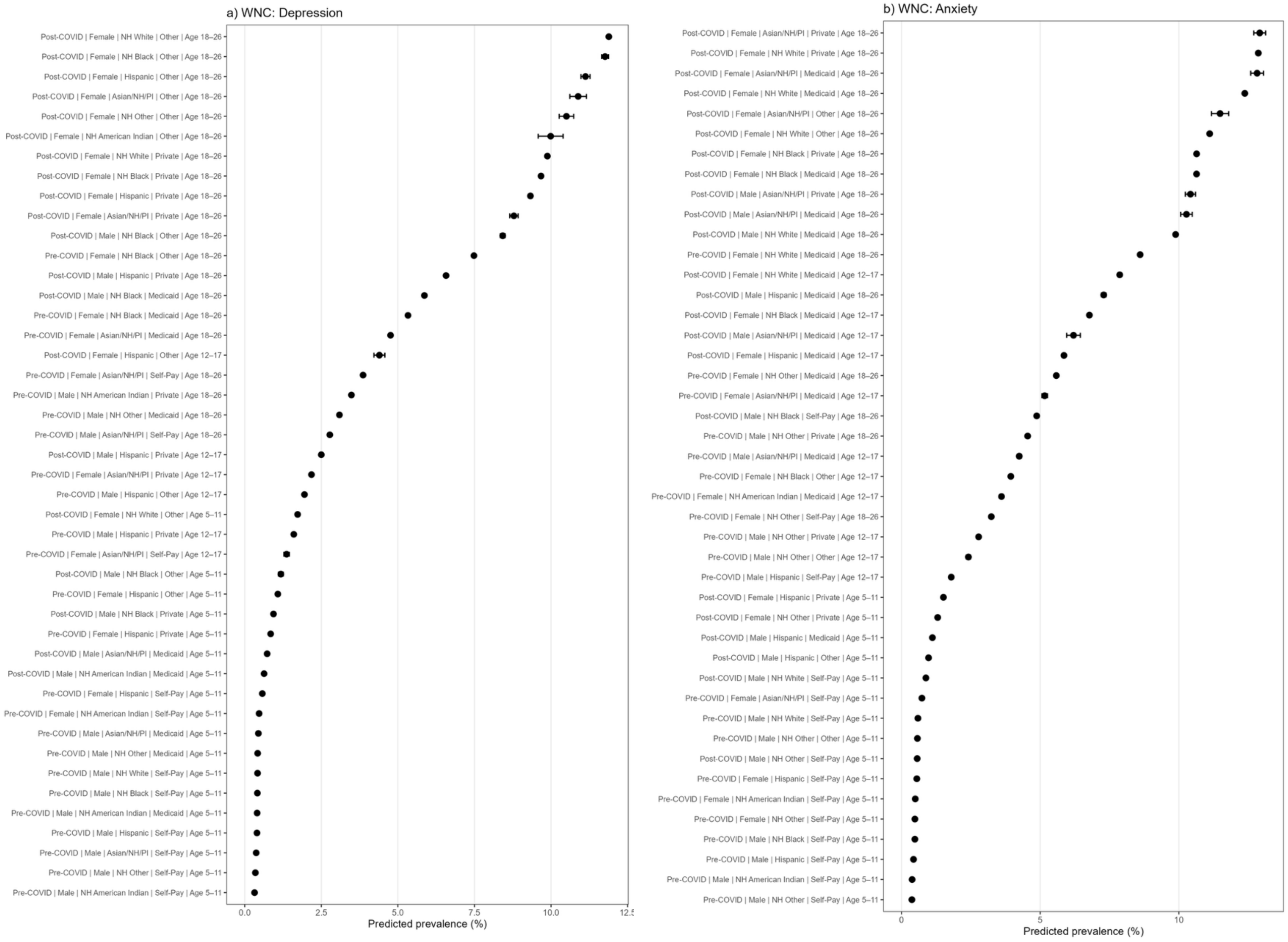

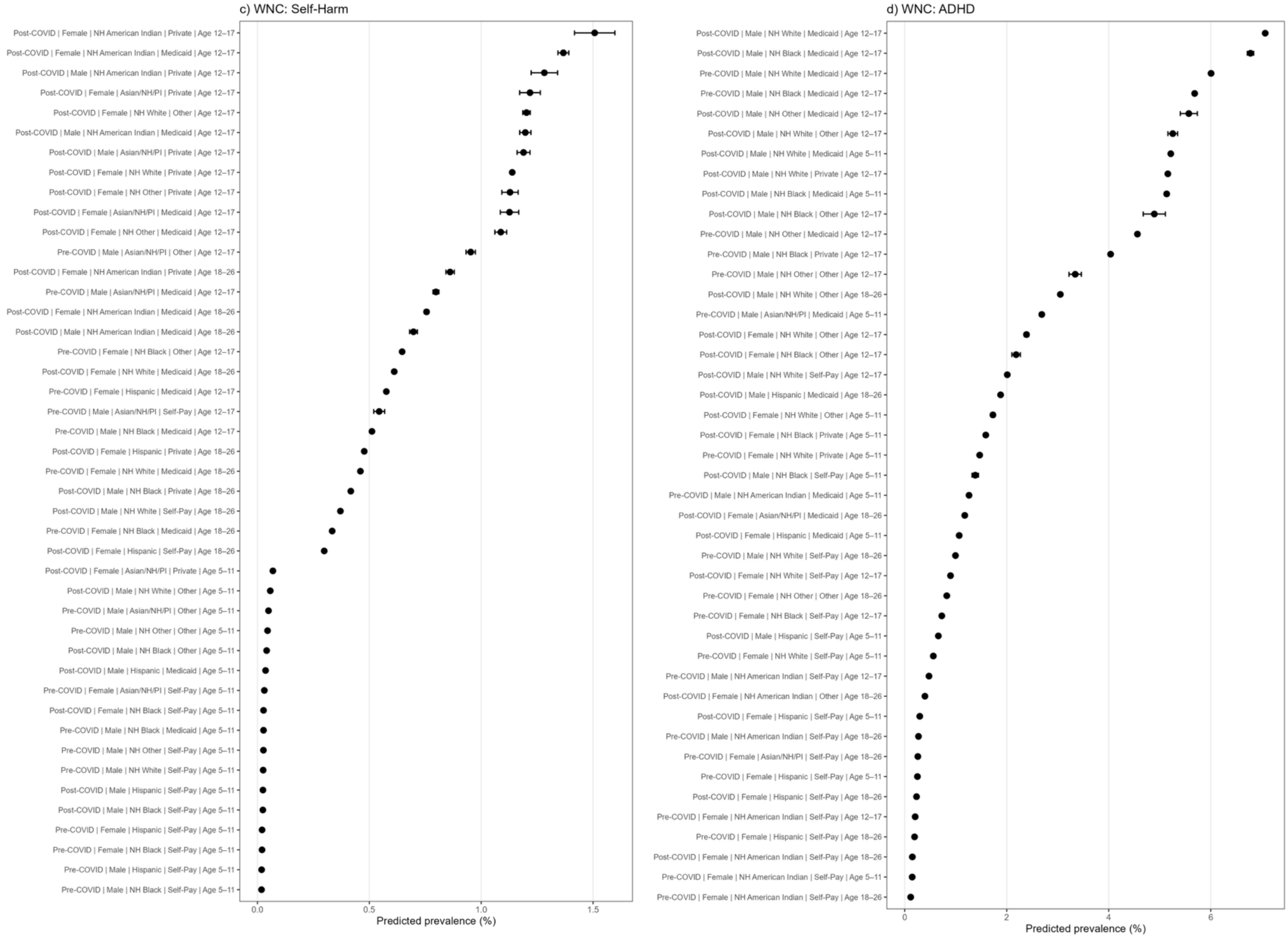
c) WNC: Self-Harm
Post-COVID | Female | NH American Indian | Private | Age 12–17
Post-COVID | Female | NH American Indian | Medicaid | Age 12–17
Post-COVID | Male | NH American Indian | Private | Age 12–17
Post-COVID | Female | Asian/NH/PI | Private | Age 12–17
Post-COVID | Female | NH White | Other | Age 12–17
Post-COVID | Male | NH American Indian | Medicaid | Age 12–17
Post-COVID | Male | Asian/NH/PI | Private | Age 12–17
Post-COVID | Female | NH White | Private | Age 12–17
Post-COVID | Female | NH Other | Private | Age 12–17
Post-COVID | Female | Asian/NH/PI | Medicaid | Age 12–17
Post-COVID | Female | NH Other | Medicaid | Age 12–17
Pre-COVID | Male | Asian/NH/PI | Other | Age 12–17
Post-COVID | Female | NH American Indian | Private | Age 18–26
Pre-COVID | Male | Asian/NH/PI | Medicaid | Age 12–17
Post-COVID | Female | NH American Indian | Medicaid | Age 18–26
Post-COVID | Male | NH American Indian | Medicaid | Age 18–26
Pre-COVID | Female | NH Black | Other | Age 12–17
Post-COVID | Female | NH White | Medicaid | Age 18–26
Pre-COVID | Female | Hispanic | Medicaid | Age 12–17
Pre-COVID | Male | Asian/NH/PI | Self-Pay | Age 12–17
Pre-COVID | Male | NH Black | Medicaid | Age 12–17
Post-COVID | Female | Hispanic | Private | Age 18–26
Pre-COVID | Female | NH White | Medicaid | Age 18–26
Post-COVID | Male | NH Black | Private | Age 18–26
Post-COVID | Male | NH White | Self-Pay | Age 18–26
Pre-COVID | Female | NH Black | Medicaid | Age 18–26
Post-COVID | Female | Hispanic | Self-Pay | Age 18–26
Post-COVID | Female | Asian/NH/PI | Private | Age 5–11
Post-COVID | Male | NH White | Other | Age 5–11
Pre-COVID | Male | Asian/NH/PI | Other | Age 5–11
Pre-COVID | Male | NH Other | Other | Age 5–11
Post-COVID | Male | NH Black | Other | Age 5–11
Post-COVID | Male | Hispanic | Medicaid | Age 5–11
Pre-COVID | Female | Asian/NH/PI | Self-Pay | Age 5–11
Post-COVID | Female | NH Black | Self-Pay | Age 5–11
Pre-COVID | Male | NH Black | Medicaid | Age 5–11
Pre-COVID | Male | NH Other | Self-Pay | Age 5–11
Pre-COVID | Male | NH White | Self-Pay | Age 5–11
Post-COVID | Male | Hispanic | Self-Pay | Age 5–11
Post-COVID | Male | NH Black | Self-Pay | Age 5–11
Pre-COVID | Female | Hispanic | Self-Pay | Age 5–11
Pre-COVID | Female | NH Black | Self-Pay | Age 5–11
Pre-COVID | Male | Hispanic | Self-Pay | Age 5–11
Pre-COVID | Male | NH Black | Self-Pay | Age 5–11
0.0
0.5
1.0
1.5
Predicted prevalence (%)
d) WNC: ADHD
Post-COVID | Male | NH White | Medicaid | Age 12–17
Post-COVID | Male | NH Black | Medicaid | Age 12–17
Pre-COVID | Male | NH White | Medicaid | Age 12–17
Pre-COVID | Male | NH Black | Medicaid | Age 12–17
Post-COVID | Male | NH Other | Medicaid | Age 12–17
Post-COVID | Male | NH White | Other | Age 12–17
Post-COVID | Male | NH White | Medicaid | Age 5–11
Post-COVID | Male | NH White | Private | Age 12–17
Post-COVID | Male | NH Black | Medicaid | Age 5–11
Post-COVID | Male | NH Black | Other | Age 12–17
Pre-COVID | Male | NH Other | Medicaid | Age 12–17
Pre-COVID | Male | NH Black | Private | Age 12–17
Pre-COVID | Male | NH Other | Other | Age 12–17
Post-COVID | Male | NH White | Other | Age 18–26
Pre-COVID | Male | Asian/NH/PI | Medicaid | Age 5–11
Post-COVID | Female | NH White | Other | Age 12–17
Post-COVID | Female | NH Black | Other | Age 12–17
Post-COVID | Male | NH White | Self-Pay | Age 12–17
Post-COVID | Male | Hispanic | Medicaid | Age 18–26
Post-COVID | Female | NH White | Other | Age 5–11
Post-COVID | Female | NH Black | Private | Age 5–11
Pre-COVID | Female | NH White | Private | Age 5–11
Post-COVID | Male | NH Black | Self-Pay | Age 5–11
Pre-COVID | Male | NH American Indian | Medicaid | Age 5–11
Post-COVID | Female | Asian/NH/PI | Medicaid | Age 18–26
Post-COVID | Female | Hispanic | Medicaid | Age 5–11
Pre-COVID | Male | NH White | Self-Pay | Age 18–26
Post-COVID | Female | NH White | Self-Pay | Age 12–17
Pre-COVID | Female | NH Other | Other | Age 18–26
Pre-COVID | Female | NH Black | Self-Pay | Age 12–17
Post-COVID | Male | Hispanic | Self-Pay | Age 5–11
Pre-COVID | Female | NH White | Self-Pay | Age 5–11
Pre-COVID | Male | NH American Indian | Self-Pay | Age 12–17
Post-COVID | Female | NH American Indian | Other | Age 18–26
Post-COVID | Female | Hispanic | Self-Pay | Age 5–11
Pre-COVID | Male | NH American Indian | Self-Pay | Age 18–26
Pre-COVID | Female | Asian/NH/PI | Self-Pay | Age 18–26
Pre-COVID | Female | Hispanic | Self-Pay | Age 5–11
Post-COVID | Female | Hispanic | Self-Pay | Age 18–26
Pre-COVID | Female | NH American Indian | Self-Pay | Age 12–17
Pre-COVID | Female | Hispanic | Self-Pay | Age 18–26
Post-COVID | Female | NH American Indian | Self-Pay | Age 18–26
Pre-COVID | Female | NH American Indian | Self-Pay | Age 5–11
Pre-COVID | Female | NH American Indian | Self-Pay | Age 18–26
0
2
4
6
Predicted prevalence (%)

S4 Figure. Predicted proportions of youth mental and behavioral health outcomes across intersectional social strata (sex, race/ethnicity, insurance type, age group, and COVID-19 period) across North Carolina. Every 10th ranked stratum combination is displayed for interpretability.

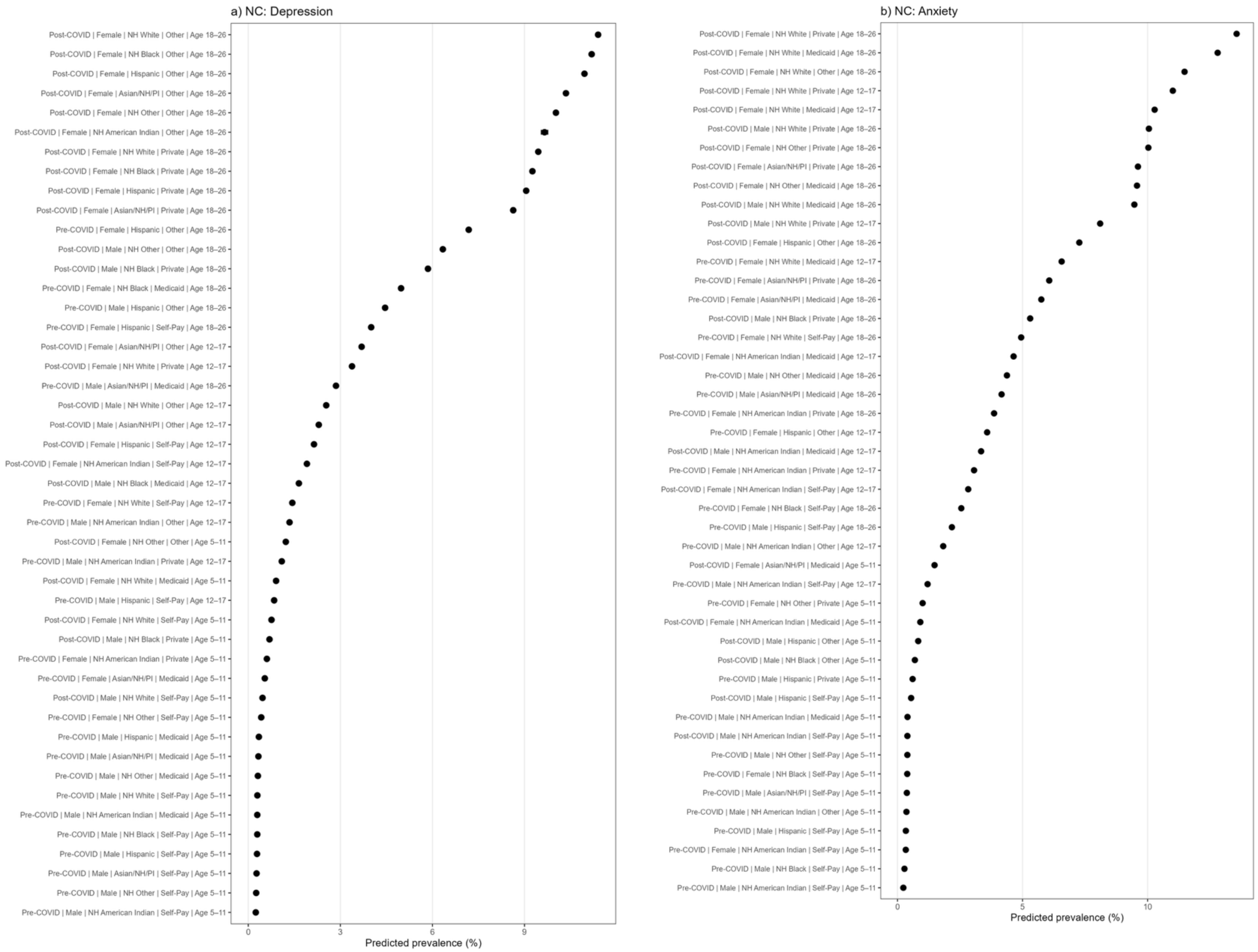

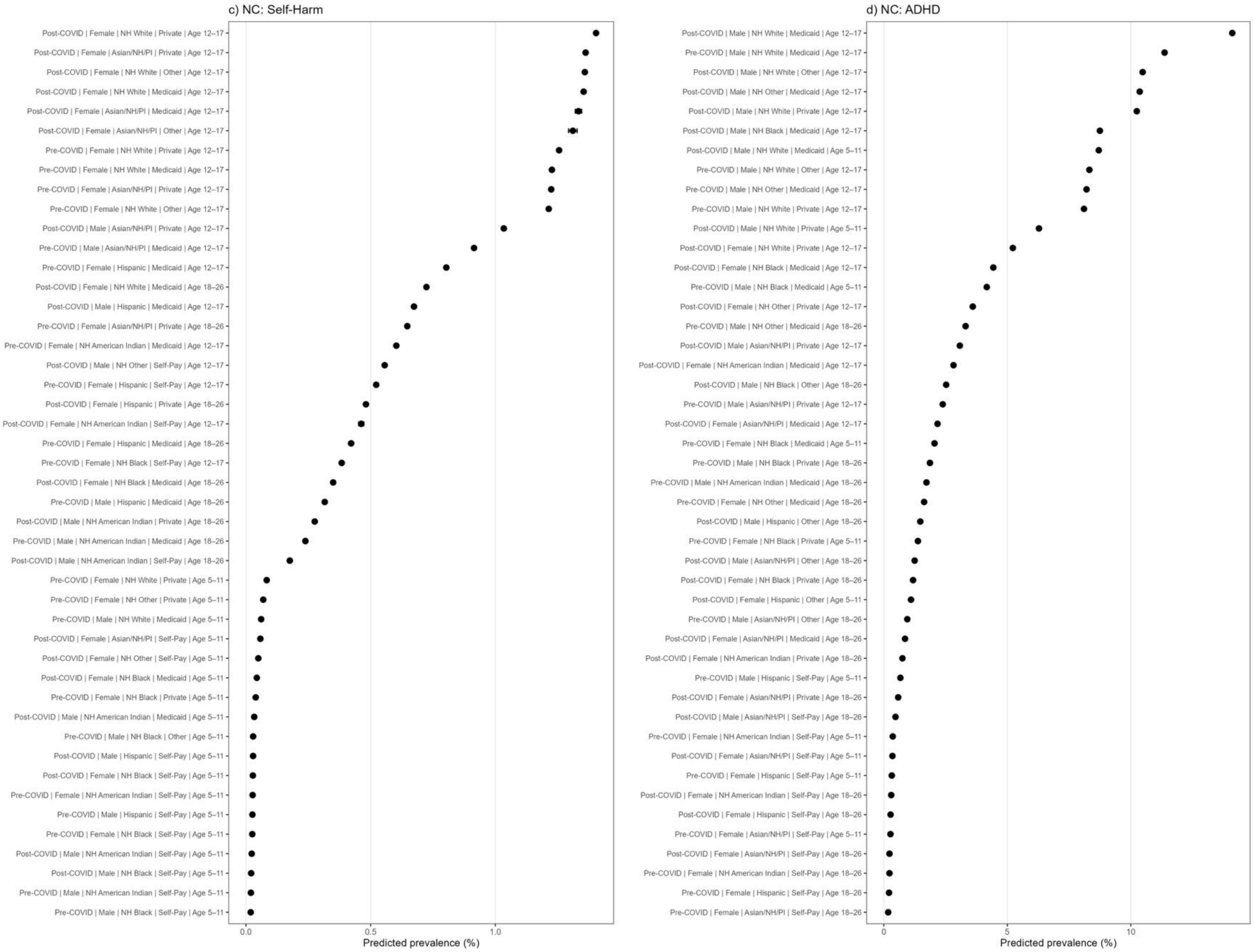

S5 Table. Sensitivity analysis: intersectional strata counts and proportions meeting observation thresholds across North Carolina and western North Carolina subregion.

Observation Per Stratum	Western North Carolina N (%)	North Carolina N (%)
100 or more	137 (56.6%)	273 (94.8%)
50 or more	173 (71.5%)	286 (99.3%)
30 or more	193 (79.8%)	288 (100.0%)
20 or more	211 (87.2%)	288 (100.0%)
10 or more	242 (100.0%)	288 (100.0%)

S6 Table. Sensitivity analysis: adjusted odds ratios (aOR) and summary statistics for youth mental and behavioral health outcomes by intersectional predictors in North Carolina and the western North Carolina subregion, March 13, 2018–Sept 30, 2022.

	**Western North Carolina aOR[a] (95%CI)[b]**				**North Carolina aOR (95%CI)**			
	**Depression**	**Anxiety**	**Self-Harm**	**ADHD**	**Depression**	**Anxiety**	**Self-Harm**	**ADHD**
COVID-19 Period								
Pre-COVID (Ref)	-	-	-	-	-	-	-	-
Post-COVID	1.12 (1.08, 1.15)	1.16 (1.12, 1.19)	1.63 (1.45, 1.83)	1.02 (0.98, 1.08)	1.16 (1.15, 1.17)	1.23 (1.22, 1.24)	1.29 (1.25, 1.33)	1.13 (1.12, 1.15)
Sex								
Male (Ref)	-	-	-	-	-	-	-	-
Female	1.37 (1.32, 1.42)	1.30 (1.26, 1.34)	1.11 (0.99, 1.25)	0.48 (0.45, 0.50)	1.52 (1.50, 1.54)	1.44 (1.42, 1.45)	1.32 (1.28, 1.37)	0.47 (0.47, 0.48)
Race/Ethnicity								
NH[c] White (Ref)	-	-	-	-	-	-	-	-
NH Black	0.77 (0.73, 0.82)	0.68 (0.65, 0.72)	0.67 (0.53, 0.85)	0.94 (0.86, 1.02)	0.49 (0.48, 0.49)	0.43 (0.43, 0.44)	0.45 (0.43, 0.47)	0.59 (0.59, 0.60)
Hispanic	0.67 (0.62, 0.73)	0.60 (0.56, 0.64)	0.72 (0.57, 0.92)	0.45 (0.40, 0.50)	0.56 (0.55, 0.57)	0.51 (0.50, 0.51)	0.63 (0.59, 0.66)	0.29 (0.29, 0.30)
NH American Indian	0.75 (0.61, 0.92)	0.67 (0.56, 0.81)	1.40 (0.82, 2.38)	0.29 (0.19, 0.45)	0.47 (0.45, 0.50)	0.45 (0.43, 0.47)	0.56 (0.47, 0.66)	0.48 (0.45, 0.51)
NH Asian/NH/PI[d]	1.37 (1.12, 1.68)	1.09 (0.90, 1.32)	0.77 (0.32, 1.85)	0.71 (0.48, 1.03)	0.86 (0.81, 0.91)	0.66 (0.62, 0.69)	1.15 (1.00, 1.33)	0.28 (0.26, 0.31)
NH Other	0.58 (0.46, 0.74)	0.56 (0.45, 0.68)	0.66 (0.31, 1.39)	0.61 (0.45, 0.85)	0.71 (0.69, 0.91)	0.66 (0.64, 0.68)	0.84 (0.77, 0.93)	0.67 (0.65, 0.70)
Insurance Payer								
Self-Pay	0.74 (0.71, 1.19)	0.77 (0.74, 0.80)	0.78 (0.65, 0.94)	0.68 (0.62, 0.74)	0.67 (0.66, 0.68)	0.71 (0.70, 0.71)	0.81 (0.77, 0.86)	0.64 (0.63, 0.66)
Medicaid	1.14 (1.09, 1.19)	1.15 (1.11, 1.19)	1.10 (0.96, 1.26)	1.72 (1.62, 1.83)	1.06 (1.05, 1.07)	1.06 (1.05, 1.07)	1.07 (1.03, 1.11)	1.88 (1.86, 1.91)

Private (Ref)	-	-	-	-	-	-	-	-
Other	1.45 (1.34, 1.56)	1.28 (1.20, 1.36)	1.65 (1.29, 2.12)	1.70 (1.51, 1.91)	1.16 (1.13, 1.18)	0.96 (0.94, 0.98)	1.22 (1.14, 1.30)	1.21 (1.18, 1.24)
Age Group								
Children (5-11) (Ref)								
Adolescents (12-17)	11.28 (10.10, 12.60)	4.63 (4.29, 4.99)	14.26 (9.92, 20.50)	1.57 (1.47, 1.68)	11.28 (10.10, 12.60)	4.63 (4.29, 4.99)	14.26 (9.92, 20.50)	1.57 (1.47, 1.68)
Young Adults (18-26)								
Variance Partition Coefficient	14.6%	4.6%	2.4%	3.5%	15.4%	2.1%	4.1%	2.4%
Proportional Change in Variance	51.7%	81.4%	91.7%	83.1%	58.3%	92.8%	87.2%	89.3%
Area Under the Curve	0.67	0.66	0.71	0.69	0.70	0.69	0.73	0.72

[a]aOR=adjused odds ratio from multivariable logistic regression models (Model 2B). each estimate is adjusted for all other predictors in the model (COVID-10 period, sex, race/ethnicity, insurance type, and age group). Reference groups indicated in the table.

[b]CI=Confidence interval

[c]NH=Non-Hispanic

[d]Asian/NH/PI=Asian/Native Hawaiian/Pacific Islander

S7 Table. Sensitivity analysis: predicted prevalence for youth mental and behavioral health outcomes: five highest- and lowest-ranked strata in North Carolina and western North Carolina subregion, March 13, 2018–Sept 30, 2022.

Rank[a]		COVID-19 Period	Sex	Race/Ethnicity	Insurance Payer	Age Group	n	Predicted Prevalence (%)
**WNC[b] - Depression**								
5 Lowest	1	Pre	Male	NH[c] Other	Self-Pay	Children (5-11)	13	0.5
	2	Post	Male	NH Other	Self-Pay	Children (5-11)	14	0.5
	3	Pre	Male	Hispanic	Self-Pay	Children (5-11)	127	0.6
	4	Pre	Male	NH White	Self-Pay	Children (5-11)	740	0.6
	5	Pre	Male	NH Black	Self-Pay	Children (5-11)	53	0.6
5 Highes*t*	*238*	Post	Female	NH Asian/PI[d]	Other	Young Adult (18-26)	19	11.5
	239	Pre	Female	NH White	Other	Young Adult (18-26)	1837	11.7
	240	Post	Female	Hispanic	Other	Young Adult (18-26)	213	11.9
	241	Post	Female	NH Black	Other	Young Adult (18-26)	214	12.9
	242	Post	Female	NH White	Other	Young Adult (18-26)	2042	13.1
**NC - Depression**								
5 Lowest	1	Pre	Male	NH American Indian	Self-Pay	Children (5-11)	249	0.3
	2	Pre	Male	NH Other	Self-Pay	Children (5-11)	677	0.4
	3	Pre	Male	NH Asian/PI	Self-Pay	Children (5-11)	139	0.4
	4	Pre	Male	Hispanic	Self-Pay	Children (5-11)	3600	0.4
	5	Pre	Male	NH American Indian	Medicaid	Children (5-11)	3289	0.4
5 Highest	284	Pre	Female	NH White	Other	Young Adult (18-26)	17802	9.7
	285	Post	Female	NH Asian/PI	Other	Young Adult (18-26)	431	10.1
	286	Post	Female	Hispanic	Other	Young Adult (18-26)	5860	10.7
	287	Post	Female	NH Black	Other	Young Adult (18-26)	18129	11.0
	288	Post	Female	NH White	Other	Young Adult (18-26)	22170	11.3
**WNC - Anxiety**								
5 Lowest	1	Pre	Male	NH Other	Self-Pay	Children (5-11)	13	0.4
	2	Post	Male	NH Other	Self-Pay	Children (5-11)	14	0.5
	3	Pre	Male	Hispanic	Self-Pay	Children (5-11)	127	0.5
	4	Post	Male	Hispanic	Self-Pay	Children (5-11)	168	0.6
	5	Pre	Female	NH Other	Self-Pay	Children (5-11)	16	0.6
5 Highest	238	Post	Female	NH White	Private	Young Adult (18-26)	14817	12.5

239	Post	Female	NH White	Other	Young Adult (18-26)	2042	12.6
240	Post	Female	NH Asian/PI	Medicaid	Young Adult (18-26)	68	13.8
241	Post	Female	NH Asian/PI	Private	Young Adult (18-26)	122	14.2
242	Post	Female	NH Asian/PI	Other	Young Adult (18-26)	19	14.8
**NC – Anxiety**							
5 Lowest 1	Pre	Male	NH American Indian	Self-Pay	Children (5-11)	249	0.3
2	Pre	Male	NH Black	Self-Pay	Children (5-11)	5135	0.3
3	Post	Male	NH American Indian	Self-Pay	Children (5-11)	115	0.4
4	Pre	Male	Hispanic	Self-Pay	Children (5-11)	3600	0.4
5	Pre	Female	NH American Indian	Self-Pay	Children (5-11)	222	0.4
5 Highest 284	Post	Female	NH White	Medicaid	Adolescent (12-17)	35281	11.1
285	Post	Female	NH White	Private	Adolescent (12-17)	40100	11.4
286	Post	Female	NH White	Other	Young Adult (18-26)	22170	12.0
287	Post	Female	NH White	Medicaid	Young Adult (18-26)	80535	13.2
288	Post	Female	NH White	Private	Young Adult (18-26)	134706	13.4
**WNC – Self-Harm**							
5 Lowest 1	Pre	Male	NH Other	Self-Pay	Children (5-11)	13	0.0
2	Pre	Male	NH Black	Self-Pay	Children (5-11)	53	0.0
3	Pre	Female	NH Black	Self-Pay	Children (5-11)	71	0.0
4	Pre	Male	Hispanic	Self-Pay	Children (5-11)	127	0.0
5	Pre	Female	NH Other	Self-Pay	Children (5-11)	16	0.0
5 Highest 238	Post	Female	NH American Indian	Private	Adolescent (12-17)	14	1.3
239	Post	Female	Hispanic	Other	Adolescent (12-17)	27	1.4
240	Post	Female	NH American Indian	Medicaid	Adolescent (12-17)	144	1.5
241	Post	Male	NH White	Other	Adolescent (12-17)	227	1.7
242	Post	Female	NH White	Other	Adolescent (12-17)	268	1.8
**NC – Self-Harm**							
5 Lowest 1	Pre	Male	NH American Indian	Self-Pay	Children (5-11)	249	0.0
2	Pre	Male	NH Black	Self-Pay	Children (5-11)	5135	0.0
3	Post	Male	NH American Indian	Self-Pay	Children (5-11)	115	0.0
4	Post	Male	NH Black	Self-Pay	Children (5-11)	5181	0.0
5	Pre	Male	Hispanic	Self-Pay	Children (5-11)	3600	0.0
5 Highest 284	Post	Female	NH White	Medicaid	Adolescent (12-17)	35281	1.4
285	Post	Female	NH Asian/PI	Private	Adolescent (12-17)	833	1.4
286	Post	Female	NH White	Other	Adolescent (12-17)	5703	1.5
287	Post	Female	NH Asian/PI	Medicaid	Adolescent (12-17)	471	1.5

288	Post	Female	NH Asian/PI	Other	Adolescent (12-17)	121	1.5
**WNC - ADHD[e]**							
5 Lowest 1	Post	Female	NH American Indian	Self-Pay	Young Adult (18-26)	82	0.2
2	Pre	Female	NH American Indian	Self-Pay	Young Adult (18-26)	140	0.2
3	Post	Female	Hispanic	Self-Pay	Children (5-11)	138	0.3
4	Post	Female	Hispanic	Self-Pay	Young Adult (18-26)	1319	0.3
5	Pre	Female	Hispanic	Self-Pay	Children (5-11)	122	0.3
5 Highest 238	Pre	Male	NH White	Private	Adolescent (12-17)	237	5.8
239	Post	Male	NH Black	Other	Adolescent (12-17)	561	6.7
240	Post	Male	NH White	Private	Adolescent (12-17)	4819	6.8
241	Pre	Male	NH Black	Other	Adolescent (12-17)	816	6.8
242	Pre	Male	NH White	Other	Adolescent (12-17)	7714	7.1
**NC - ADHD**							
5 Lowest 1	Pre	Female	NH Asian/PI	Self-Pay	Young Adult (18-26)	996	0.2
2	Post	Female	NH Asian/PI	Self-Pay	Young Adult (18-26)	805	0.3
3	Pre	Female	Hispanic	Self-Pay	Young Adult (18-26)	24056	0.3
4	Post	Female	Hispanic	Self-Pay	Young Adult (18-26)	26103	0.3
5	Pre	Female	NH Asian/PI	Self-Pay	Children (5-11)	111	0.3
5 Highest 284	Pre	Male	NH White	Other	Adolescent (12-17)	3559	9.8
285	Post	Male	NH Other	Medicaid	Adolescent (12-17)	1827	10.0
286	Post	Male	NH White	Other	Adolescent (12-17)	3925	10.2
287	Pre	Male	NH White	Medicaid	Adolescent (12-17)	45512	13.0
288	Post	Male	NH White	Medicaid	Adolescent (12-17)	26370	13.6

[a]Strata were defined by COVID-19 period, sex, race/ethnicity, insurance type, and age group, with the five lowest- and five highest-ranked strata identified separately for each outcome.

[b]WNC=Western North Carolina

[c]NH=Non-Hispanic

[d]Asian/PI=Asian/Pacific Islander

[e]ADHD=Attention-deficit/hyperactivity disorder
